# Pediatric Case Report and Overview of Autochthonous Tick-Borne Encephalitis, Belgium

**DOI:** 10.3201/eid3109.250093

**Published:** 2025-09

**Authors:** Justine De Langhe, Jo Sourbron, Robbe Van Herreweghe, Marjan van Esbroeck, Koen Vercauteren, Tessa de Block, Jasmine Coppens, Daan Jansen, Dorien Van den Bossche, Veerle Staelens, Sarah De Schryver, Jos Van Acker

**Affiliations:** Author affiliations: Ghent University Hospital, Ghent, Belgium (J. De Langhe); Ghent University Faculty of Medicine and Health Sciences, Ghent (J. De Langhe, R. Van Herreweghe); University Hospital KU Leuven, Leuven, Belgium (J. Sourbron); Ghent University Hospital Center for Medical Genetics, Ghent (J. Sourbron); Institute of Tropical Medicine Antwerp, Antwerp, Belgium (M. van Esbroeck, K. Vercauteren, T. de Block, J. Coppens, D. Jansen, D. Van den Bossche); AZ Sint-Lucas Gent, Ghent (V. Staelens, S. De Schryver, J. Van Acker);

**Keywords:** tick-borne encephalitis, tick-borne encephalitis virus, viruses, vector-borne infections, meningitis/encephalitis, ticks, Belgium

## Abstract

Prevalence of tick-borne encephalitis (TBE) is increasing in much of Europe. In May 2024, an autochthonous pediatric case of TBE was diagnosed in a 6-year-old girl in Belgium. Clinicians should recognize the symptoms and signs of TBE infections and consider this disease in patients with unexplained neurologic symptoms, regardless of travel history.

Tick-borne encephalitis (TBE) is a disease of the central nervous system (CNS) caused by TBE virus (TBEV). TBEV is endemic in regions from Europe to the Far East, where ixodid ticks act as vectors (*1*–*3*). TBEV has 3 main subtypes: European, Siberian, and Far Eastern (*2*–*4*). 

Since 1973, TBE incidence has increased by nearly 400% in Europe, excluding Portugal and Belgium. TBE is mainly transmitted from late spring to early autumn, and spread is linked to global warming (*1*–*3*). TBEV infection can also occur by consumption of unpasteurized milk products from infected livestock (*1*,*3*).

Clinical course and outcomes vary by TBEV subtype. The European subtype often causes a biphasic illness. Up to 10% of TBE patients develop pareses from myelitis, and the mortality rate is 0.5%–2%. Symptoms begin 8 days after tick bite (incubation range 4–28 days) with a nonspecific febrile illness (viremic phase), which resolves before potentially progressing to CNS inflammation 2–8 days later (neurotrophic phase). Neurologic symptoms include meningitis and meningoencephalitis, typically lasting 7–10 days (*1*,*3*). Preventive measures are essential, because no effective treatment exists (*1*).

According to the literature available through August 2024, in Belgium, 8 nonautochthonous (*5*) and 3 autochthonous (*6*) TBE cases had occurred in adults. We report an autochthonous pediatric TBE case in Belgium and compare that case to the 3 autochthonous TBE cases in adults.

A 6-year-old girl was brought for care with a 6-day history of fever, diarrhea, and myalgia. She had returned from Thailand 3 weeks earlier and engaged in several outdoor activities after her return to Belgium. A clinical examination did not identify a cause for the fever. Blood tests showed mild thrombocytopenia, leukopenia, and elevated creatine kinase (CK) (Table). An infectious serology search focused on common infections in Thailand. Results of testing of a urine sample and nasopharyngeal swab specimen were negative. A stool sample showed the presence of *Salmonella enterica* serovar Bareilly and *Campylobacter jejuni*; azithromycin was initiated for 3 days. During her 5-day hospital stay, the patient showed clinical improvement, and her fever resolved.

**Table:** Details of autochthonous tick-borne encephalitis cases in a child compared with 3 previous cases in adults, Belgium*

Characteristic	Patient no. from 2021 report (*6*)			Case report from 2024 (this study)
	1	2	3	
Age of onset, y/sex	48/F	59/M	58/M	6/F
Tick bite†	2 weeks before symptom onset	2 weeks before symptom onset	Multiple tick bites in the weeks before symptom onset	No observed tick bite, but increased outdoor activities
Likely site of tick bite, postal code (province)‡	Oostkamp, 8020 (West Flanders)	Lille, 2275 (Antwerp)	Wanze, 4520 (Liège)	Evergem, 9940 (East Flanders)
Signs/symptoms				
During first (viremic) phase	Myalgia, fever	Fever, fatigue, myalgia, headache	Dyspnea, cough, fever	Recurrent fever, diarrhea, anorexia, arthralgia (ankles, hands and wrists), myalgia, ophthalmalgia, cervicalgia
During second (neurotrophic) phase	Asthenia, tremor, drowsiness, fever, peripheral facial palsy, brachial weakness, nuchal rigidity	Fever, fatigue, myalgia, headache, paraparesis, signs of meningitis, severe motor polyradiculitis	Recurrent fever, severe and persistent headaches, weakness, diarrhea, anorexia	Spiking fever, photophobia, vomiting, agitated behavior, fatigue, myalgia, arthralgia, diarrhea
At follow-up (time)	Weakness of right arm, loss of cognitive function, inability to concentrate, fatigue, tremor (≈2 mo)	Improved motor skills 9 mo after hospitalization (wheelchair at discharge) (9 mo)	Occasional headaches, otherwise recovered (≈2 mo)	No residual symptoms (6 mo)
Sample type (no. days after symptom onset)	Serum (5), CSF (6)	Serum (20), CSF (18)	Serum (2), serum (18)	Serum (5), serum (17), CSF (17), serum (19), serum (26)
Flavivirus IFA serum (no. days after symptom onset)	Serum (5): IgM, TBEV+; IgG, TBEV+	Serum (20): IgM, TBEV+; IgG, TBEV+	Serum (2): IgM, TBEV–; IgG, TBEV–. Serum (18): IgM, TBEV+; IgG, TBEV+	Serum (5): IgM, TBEV–; IgG, TBEV–. Serum (17): IgM, TBEV+ (>1/80); IgG, TBEV+ (>1/80). Serum (26): IgM, TBEV+ (>1/80); IgG, TBEV+ (>1/80)
Flavivirus IFA CSF (days after symptom onset)	CSF (6): IgM, TBEV+; IgG, TBEV+	CSF (18): IgM, TBEV+; IgG, TBEV+	ND	CSF (17): IgM, TBEV+; IgG, ND (sample too small for both Ig types)
PRNT_90_ titer (no. days after symptom onset)	Serum (5): 1:25; CSF (6): ND	Serum (20): 1:60; CSF (18): ND	Serum (2): ND; serum (18): 1:194	Serum (26): 1:204
rRT-PCR (no. days after symptom onset)	Serum (5): ND; CSF (6): ND	Serum (20): ND; CSF (18): ND	Serum (2): +; serum (18): ND	Serum (5): + (Ct 36.27); serum (17): ND; CSF (17): ND; serum (19): ND; serum (26): ND
TBEV RNA sequencing (serum)	ND	ND	ND	European subtype TBEV

*Additional case characteristics are provided in Appendix Table 2. CSF, cerebrospinal fluid; Ct, cycle threshold; IFA, indirect fluorescent antibody; ND, not determined; PRNT_90_, 90% plaque reduction neutralization test; rRT-PCR, real-time reverse transcription PCR; TBEV, tick-borne encephalitis virus; +, present/positive; –, absent/negative. †Early removal of the tick might not prevent encephalitis ​(*4*)​. Approximately 30% of cases occur without a reported tick bite ​(*2*,*4*)​. ‡Locations show no clear proximity (Appendix Figure 3).

Four days after discharge, the girl was readmitted because of recurrent fever for 1 day and arthralgia. Additional blood results showed no anomalies. On day 4 of readmission, meningeal signs appeared, and blood tests showed leukocytosis. Extensive imaging showed no anomalies. A lumbar puncture showed cerebral spinal fluid (CSF) leukocytosis, prompting intravenous cefotaxime. Extensive serology testing was performed. After 7 days of intravenous cefotaxime treatment, TBEV IgM was detected in CSF. The patient, whose symptoms resolved, was discharged. Follow-up consultations indicated favorable recovery without residual symptoms. A brain magnetic resonance imaging scan showed no cerebral injuries.

We compared the clinical course of this patient to those of the 3 previous autochthonous TBEV cases in Belgium (Table). We noted no geographic links between those 3 cases and the pediatric case we report. In all cases, a biphasic course was observed. In 2 of the previous cases, long-term neurologic sequelae were documented several months postinfection; however, we did not observe such sequelae in our case. In cases with persistent neurological deficits, paresis was already evident during the neurotrophic phase, consistent with the 10% of TBE patients who develop paresis as a result of myelitis.

We observed a biphasic course, observed in 75% of TBE cases (*1*–*3*), in this patient. Serum CK levels were also elevated (*4*). Some TBE patients have myalgia/myositis, and up to one third have elevated serum CK levels (*7*), yet the clinical relevance is still unclear. After the patient had a symptom-free interval, fever recurred, and meningitis was confirmed. TBEV IgM was detected in serum and CSF. Testing for Japanese encephalitis was initiated because of the patient’s travel history; TBEV testing was included in that panel.

Given the incubation range of 4–28 (median 8) days (*2*,*3*), we cannot exclude possible TBEV infection in Thailand. However, no TBE cases have been reported in Thailand (*8*). Moreover, sequencing of the TBEV RNA from a serum sample identified the European subtype, suggesting infection acquired in Europe (Appendix). Although no tick bite was reported for this patient, her increased outdoor activities posed a substantial risk; tick bites go unnoticed in about one third of TBE cases (*3*).

A rare transmission route of TBEV (1% of all cases) is through consuming unpasteurized milk from infected livestock (*9*). This patient consumed unpasteurized cow’s milk >2 weeks before symptoms appeared; however, the Federal Agency for Food Chain Safety in Belgium investigated the identified producer’s milk and found no TBEV RNA.

Of note, the patient’s dog had recently been euthanized because of a suspected stroke. Neurologic signs such as ataxia, plegia/paresis, cranial nerve deficits, and seizures have been described in TBEV-infected dogs (*10*). However, postmortem investigations were not performed because the animal was cremated.

Because most (70%–98%) TBEV infections are asymptomatic, prevalence of TBEV infections is presumably underestimated (*4*,*6*). Clinicians should recognize the signs and symptoms of TBEV infection and consider TBE in patients with unexplained neurologic symptoms, particularly a biphasic course (*2*).

Appendix Additional information for pediatric case report and overview of autochthonous tick-borne encephalitis, Belgium.
