# Supplementary material for: Pediatric Case Report and Overview of Autochthonous Tick-Borne Encephalitis, Belgium
Source: Emerg Infect Dis. 2025 Sep;31(9):1868–70. doi: 10.3201/eid3109.250093 (PMC12407206; doi:10.3201/eid3109.250093)
Supplement: Appendix — Additional information for pediatric case report and overview of autochthonous tick-borne encephalitis, Belgium. [file 25-0093-Techapp-s1.pdf]

Article DOI: https://doi.org/10.3201/eid3109.250093

EID cannot ensure accessibility for supplementary materials supplied by authors. Readers who have difficulty accessing supplementary content should contact the authors for assistance.

Pediatric Case Report and Overview of Autochthonous Tick-Borne Encephalitis, Belgium

Appendix

Additional serology and sequencing data

TBEV sequencing

Methods

Sample preparation and sequencing

RNA was extracted from a clinical serum sample, and the Sequence-Independent Single-Primer Amplification (SISPA) method was employed to increase the amount of genetic material for sequencing. SISPA includes a reverse transcription step that uses tagged random nine-base primers. The tag enables subsequent amplification with a primer complementary to the universal tag. The Twist Comprehensive Viral Research panel was then used to capture viral sequences. Following ONT adaptor ligation with the Ligation Sequencing Kit V14 (SQK-LSK114), the library was prepared for sequencing on a MinION R10.4 flow cell.

Data analysis and viral detection

Raw sequencing data was stored in FAST5 files and directly used as an input for our in-house viral metagenomics pipeline. In brief, FAST5 files were converted to FASTQ files by using Guppy and filtered based on quality and read length with Fastp. Reads mapped to the human genome by using Minimap2 were depleted. The remaining reads were mapped against a viral database with MetaMaps for viral taxonomic classification. The reference sequence of the matching viral taxon was then used to generate a consensus sequence by using Minimap2 followed by Medaka, iVar and homopolish.

Phylogenetic analysis

TBEV genomes (n = 58) were extracted from the NCBI Virus database (Appendix Table 1), with filters applied for Virus/Taxonomy (Tick-borne encephalitis virus taxid 11084), complete nucleotide sequences, and human as the host. This set of TBEV genomes was aligned by MAFFT (v 7.525) together with the consensus genome generated by our in-house pipeline. A maximum-likelihood (ML) phylogenetic tree was inferred from the alignment using IQ-TREE (v 2.3.6) with the MFP model, which automatically selects the best-fit substitution model using ModelFinder. Branch support for the ML phylogeny was assessed with 1000 standard non-parametric bootstrap analyses. Interactive tree of life (iTOL) was used to visualize the resulting tree.

Results

Viral detection

Our in-house viral metagenomics pipeline identified viral reads derived from the clinical sample matching solely to TBEV (n = 113492). The completeness of the generated consensus sequence was 52% with a vertical coverage of 5191 (Appendix Figure 1).

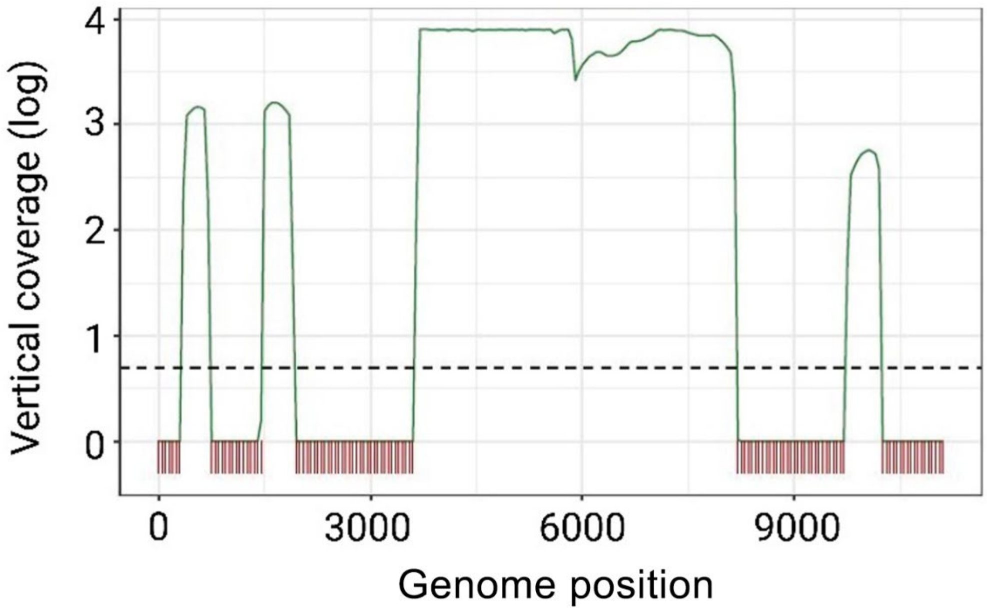

Appendix Figure 1. Coverage plot of TBEV reads identified in the sequence data from the clinical sample. On the x-axis every position of the TBEV reference genome with corresponding vertical coverage (depth) on the y-axis in log scale. Red vertical lines indicates gaps in the consensus genome.

Phylogenetic analysis

A phylogenetic approach was used to characterize the TBEV subtype of the virus detected in the clinical sample. The consensus genome clustered with European subtypes, indicating that the clinical sample contained the European subtype of TBEV (Appendix Figure 2).

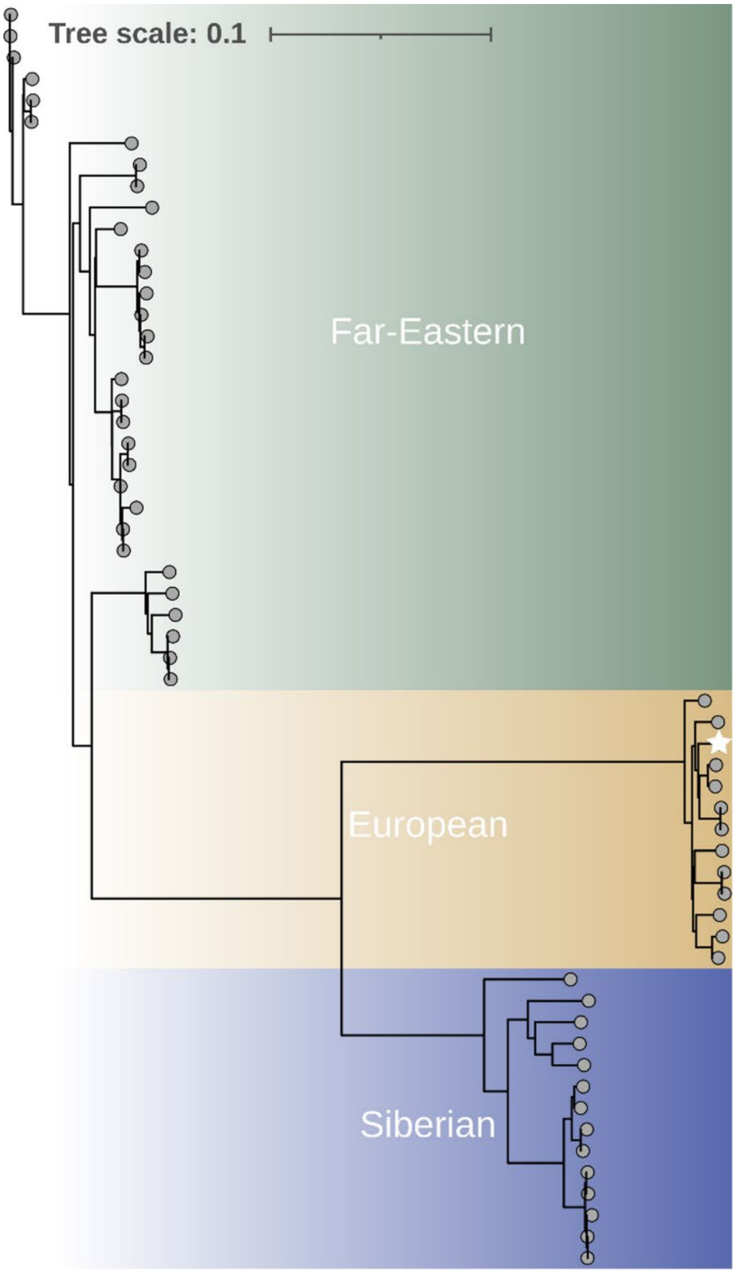

Appendix Figure 2. Unrooted maximum likelihood phylogeny tree based on alignment of publicly available genome sequences (gray dots) and the consensus sequences derived from the clinical sample (white star). Clusters are annotated based on the TBEV subtype (Far-Eastern, European and Siberian).

Appendix Table 1. NCBI number of TBEV genomes extracted from NCBI virus platform including year of isolation and country. Subtypes are determined based on the tree results if not specified in the NCBI database

NCBI_ID	TBEV subtype	Country	Year of isolation
KJ922515.1	European	Czech	NA
KJ922513.1	European	Czech	NA
KJ922516.1	European	Czech	NA
KJ922512.1	European	Czech	NA
MG589937.1	European	Finland	NA
OQ435379.1	European	Estonia	1987
MT581212.1	European	Sweden	2019
MK560446.1	European	Russia	NA
MK562430.1	European	Russia	1967
KU885457.1	European	Russia	NA
KJ000002.1	European	Russia	1951
KJ922514.1	European	Czech	NA
GU121642.1	Far-Eastern	Russia	2008
FJ402885.1	Far-Eastern	Russia	1985
JQ650522.1	Far-Eastern	People’s Republic of China	2001
JQ650523.1	Far-Eastern	People’s Republic of China	1953
JF316708.1	Far-Eastern	People’s Republic of China	2010
JF316707.1	Far-Eastern	People’s Republic of China	2010
KJ739729.1	Far-Eastern	Russia	2008
FJ997899.1	Far-Eastern	Russia	1990
PP947713.1	Far-Eastern	Russia	NA
PP708890.1	Far-Eastern	Russia	1999
GQ228395.1	Far-Eastern	Russia	1997
KU761567.1	Far-Eastern	Russia	1958
KU761574.1	Far-Eastern	Russia	1958
KU761572.1	Far-Eastern	Russia	1958
KU761575.1	Far-Eastern	Russia	1958
KU761569.1	Far-Eastern	Russia	1960
FJ906622.1	Far-Eastern	Russia	1987
KU761576.1	Far-Eastern	Russia	1937
MT671300.1	Far-Eastern	Russia	1962
KF951037.1	Far-Eastern	Russia	1966
JF819648.2	Far-Eastern	Russia	1937
MN115817.1	Far-Eastern	Russia	1969
MT671301.1	Far-Eastern	Russia	1969
MT671302.1	Far-Eastern	Russia	1957
HQ901303.1	Far-Eastern	Russia	1992
PP708887.1	Far-Eastern	Russia	1991
HQ901367.1	Far-Eastern	Russia	2010
HQ901366.1	Far-Eastern	Russia	2009
FJ402886.1	Far-Eastern	Russia	1973
PP937585.1	Far-Eastern	Russia	1991
PP937586.1	Far-Eastern	Russia	1991
PP937587.1	Far-Eastern	Russia	1992
MG589939.1	Siberian	Finland	2015
MN115819.1	Siberian	Russia	1964
MN115820.1	Siberian	Russia	1966
KC414090.1	Siberian	Russia	1999
MT670183.1	Siberian	Russia	1963
MN115818.1	Siberian	Russia	1965
MT670184.1	Siberian	Russia	1966
MH645612.1	Siberian	Russia	2003
MN114635.1	Siberian	Russia	2018
MF043955.1	Siberian	Russia	NA
OQ565596.1	Siberian	Russia	2013
MF043954.1	Siberian	Russia	NA
KF644245.1	Siberian	Russia	2013
MF043953.1	Siberian	Russia	NA

TBEV RT-PCR

TBEV RT-PCR was done using real-time reverse transcription PCR, adapted from (*1*).

Serology

TBEV-/JE-serology

Detection of TBEV-/JE-antibodies was performed using an immunofluorescence assay (Flavivirus Profile 2, Euroimmun AG, Lübeck, Germany).

Dengue serology

Detection of dengue IgM antibodies was performed with the Dengue Virus IgM Capture Dx Select (Focus Diagnostics, Cypress, CA, USA). Dengue IgG antibodies were detected with the Dengue Virus IgG Dx Select (Focus Diagnostics).

Belgian autochthonous Tick-Borne Encephalitis cases

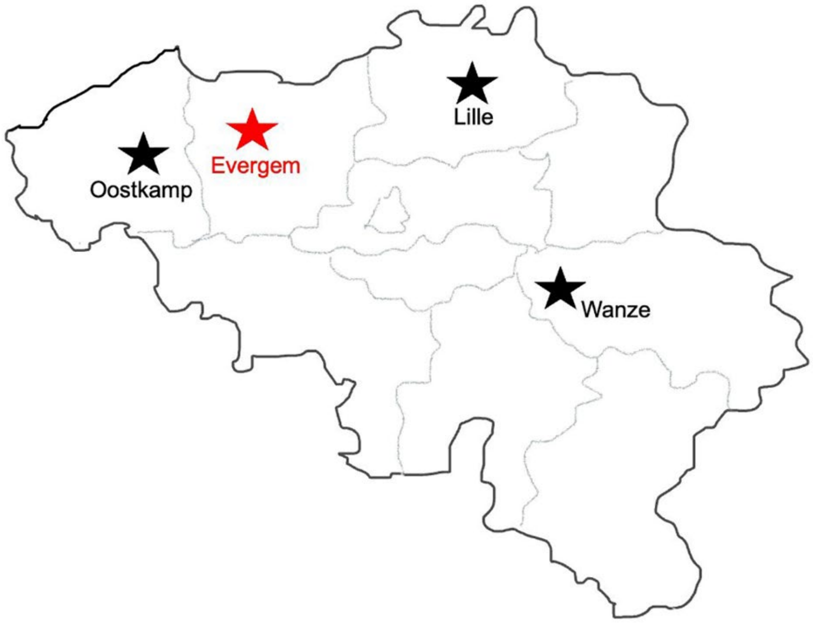

Appendix Figure 3. Four autochthonous Tick-Borne Encephalitis cases in Belgium depicted on a geographic map of Belgium. We note no geographic link between our case (red star) and the previous 3 confirmed cases (black stars).

Reference

1. Stoefs A, Heyndrickx L, De Winter J, Coeckelbergh E, Willekens B, Alonso-Jiménez A, et al. Autochthonous cases of tick-borne encephalitis, Belgium, 2020. Emerg Infect Dis. 2021;27:2179–82. PubMed

Appendix Table 2. Additional details of autochthonous tick-borne encephalitis cases in a child in Belgium compared with 3 previous cases in adults*

	Patient no. from 2021 report (*6*)			
Characteristic	1	2	3	Case report from 2024 (this study)
Travel history	None	None	None	Thailand, 3 wk before illness
Biphasic course	ND	ND	+	+
Signs/symptoms				
Fever	+	+	+	+
Headache	–	+	+	+
Fatigue	+	+	–	+
Irritability	–	–	–	+
Meningeal signs	+	+	–	+
Disturbance of consciousness	+	+	–	–
Paresis	Facial palsy, brachial weakness	Motor polyradiculitis causing motor dysfunction	–	–
Myalgia	+	+	–	+
Cognitive impairment	+	–	–	–
Diarrhea	–	–	+	+
Tremor	+	–	–	–
Additional investigations				
Laboratory findings†	ND	ND	ND	First phase: leukocytes, 1,800/µL; neutrophils, 680/µL; lymphocytes, 980/µL; platelets, 115,000/µL; CK, 423 U/L; CRP, <1 mg/L. Second phase: leukocytes, 20,500/µL; neutrophils, 15,590/µL; lymphocytes, 3,650/µL; platelets, 395,000/µL; CK, 51 U/L; CRP, <1 mg/L
Serology	ND	ND	ND	Negative for malaria, dengue, chikungunya, Zika, *Borrelia*, CMV, EBV, *Brucella*, *Leishmania*, *Leptospira*, JEV, WNV
Nasopharyngeal sample	ND	ND	ND	Negative for influenza A/B, COVID-19, RSV, adenovirus, enterovirus, metapneumovirus, parainfluenzavirus 1/2/3/4, *Legionella pneumophila*, *Chlamydophila pneumoniae*, *Chlamydophila psittaci*
Urine sample	ND	ND	ND	–
Stool sample	ND	ND	ND	Positive for *Salmonella enterica serovar* Bareilly, *Campylobacter jejuni*
Rheumatologic testing	ND	ND	ND	Negative for ANF, CTD screening
Imaging	Brain MRI: demyelinating lesions and encephalopathy	Negative MRI, PET	Negative PET	Negative abdominal ultrasound, bone scan, brain CT; brain MRI: aspecific enhancement of facial and vestibular nerves without clinical correlate
EEG	Diffuse slow activity	ND	ND	ND
Cardiac investigations	ND	ND	Negative TEE	Negative TEE
CSF leukocytes§	37 cells/µL	371 cells/µL	ND	106 cells/µL; lymphocytes, 69.8%; monocytes, 19.8%; segments, 10.4%; eosinophils, 0%
CSF protein§	ND	ND	ND	65 mg/dL

*+, present/positive; –, absent/negative; ANF, antinuclear factor; CK, creatine kinase; CMV, cytomegalovirus; CRP, C-reactive protein; CSF, cerebrospinal fluid; CT, computed tomography; CTD, connective tissue disease; EBV, Epstein-Barr virus; EEG, electroencephalogram; MRI, magnetic resonance imaging; ND, not determined; PET, positron emission tomography; RSV, respiratory syncytial virus; TEE, transesophageal echocardiogram; TTE, transthoracic echocardiogram.

†To prevent an excess of data, only deviating and/or clinically relevant biochemical markers are listed. Reference values: leukocytes 4 500–13,500/µL; neutrophils, 1,500–8,000/µL; lymphocytes 1,500–6,800/µL; platelets, 164,000–369,000/µL; CK, 20–180 U/L; CRP, <5 mg/L; CSF leukocytes, 0–5/µL; CSF protein, 15–40 mg/dL
